# Pancreatic-carcinoma-cell-derived pro-angiogenic factors can induce endothelial-cell differentiation of a subset of circulating CD34+ progenitors

**DOI:** 10.1186/1479-5876-11-314

**Published:** 2013-12-17

**Authors:** Barbara Vizio, Fiorella Biasi, Tiziana Scirelli, Anna Novarino, Adriana Prati, Libero Ciuffreda, Giuseppe Montrucchio, Giuseppe Poli, Graziella Bellone

**Affiliations:** 1 Department of Medical Sciences, University of Turin, Turin, Italy; 2 Department of Clinical and Biological Sciences, University of Turin, Turin, Italy; 3 Department of Medical Oncology, Azienda Ospedaliera Città della Salute e della Scienza di Torino, Turin, Italy

**Keywords:** CD34^+^ cells, Endothelial progenitor cells, Pancreatic carcinoma, H6c7 cells, Angiogenic growth factors, Tumor neoangiogenesis

## Abstract

**Background:**

CD34^+^ progenitor cells comprise both hematopoietic and endothelial progenitor cells. Recent studies suggest that circulating endothelial progenitor cells are recruited into the angiogenic vascular system of several cancers, including pancreatic carcinoma, and that they correlate with clinical progress. However, whether endothelial progenitor cell mobilization occurs in response to cytokine release by tumor cells is still unclear.

**Methods:**

The chemotactic- and/or differentiating-activities of the poorly-differentiated pancreatic carcinoma cell line PT45, and of the immortal H6c7 cell line, a line of near-normal pancreatic duct epithelial cells, on endothelial progenitor cells were investigated *in vitro* using circulating CD34^+^ as model.

**Results:**

The study showed that Vascular Endothelial Growth Factor produced by PT45 cells and, at lesser extent, by H6c7 cells, predominantly chemoattract peripheral blood CD34^+^ expressing the type 2 relative receptor. Addition of PT45-conditioned medium to CD34^+^ cells, cultured under conditions supporting myeloid cell development, diverted the differentiation of a subset of these progenitor cells into cells expressing endothelial cell markers, such as CD146, CD105, VE-cadherin and von Willebrand Factor-related antigen. Moreover, these endothelial-like cells formed capillary networks *in vitro,* chiefly through the release of Angiopoietin-1 by PT45 cells.

**Conclusions:**

The results demonstrate that pancreatic-carcinoma cells potentially attract circulating endothelial progenitor cells to the tumor site, by releasing high levels of pro-angiogenic factors such as Vascular Endothelial Growth Factor and Angiopoietin-1, and may direct the differentiation of these cell subsets of the CD34^+^ cell population into endothelial cells; the latter cells may become a component of the newly-formed vessels, contributing to angiogenesis-mediated tumor growth and metastasis.

## Background

It is well known that, in order to develop in size and metastatic potential, tumors must make an "angiogenic switch"; they achieve this by perturbing the local balance of pro-angiogenic and anti-angiogenic factors [1]. When positive angiogenic regulators overcome the effect of negative angiogenic molecules, the tumor acquires angiogenic capability, leading to the formation of new blood vessels.

Tumor cells may induce angiogenesis directly, by releasing pro-angiogenic factors, and/or indirectly, by attracting inflammatory cells that, in turn, release angiogenic stimuli [2]. The number of factors known to be capable of eliciting an angiogenic response is steadily increasing. Vascular Endothelial Growth Factor (VEGF) is considered a master regulator of the angiogenic cascade, and is thought to promote endothelial cell differentiation, migration, proliferation, and survival [3]. The finding that a number of malignant human tumors, including lung, breast, gastrointestinal tract, ovary, and colon, produce VEGF [4-8], and that the inhibition of VEGF-induced angiogenesis significantly inhibits tumor growth *in vivo*[9], point to its possessing clinical significance in tumor growth.

Pancreatic carcinoma is a biologically-aggressive tumor that has an early propensity to spread locally and metastasize distally. While not grossly vascular, this tumor often exhibits enhanced foci of endothelial cell proliferation, and over-expresses multiple pro-angiogenic factors [10,11]. In particular, a positive correlation between blood vessel density, tumor VEGF-A isoform levels, and disease progression has been reported in pancreatic carcinoma [12-14].

The formation of new capillaries to provide an oxygen supply for tumors was, until recently, believed to be mediated by the sprouting or co-option of pre-existing neighboring vessels [15].

However, increasing evidence now suggests that circulating endothelial progenitor cells (EPC), normally involved in maintaining vascular integrity, can also home in to the tumor site and contribute to the *de novo* formation of blood vessels [16].

In previous studies we found that VEGF expression in pancreatic carcinoma cell lines is both high and inversely correlated with differentiation status [10]. Moreover, EPC and VEGF-A plasma levels were found to be significantly elevated in the blood of pancreatic carcinoma patients, to be positively associated with disease stage, and inversely associated with overall survival [17]. These finding suggest that microenvironmental conditions favoring mobilization of EPC, which are key contributors to the early steps in neoplastic vascularization [18], might enable the tumor to grow and metastasize faster. However, there is ongoing debate about the distribution, contribution, origin, and differentiation of EPC in tumor vasculogenesis.

The present research aimed to investigate the ability of pancreatic carcinoma cells to attract and skew the differentiation of CD34^+^ progenitor cells toward endothelial cells, by releasing pro-angiogenic factors. We show that PT45 cells, as normal pancreatic ductal epithelial cells, promote the recruitment of CD34^+^ cells. Moreover, when cultured under conditions that facilitate myeloid-cell development, CD34^+^ cells are instead redirected by the tumor to differentiate into endothelial cells. The resulting cells resemble endothelial cells phenotypically, as well as functionally, as is shown by the fact they can be stimulated to reorganize into cord structures. Tumor-derived VEGF contributed significantly to the chemoattractant activity, whereas Angiopoietin (Angio)-1 chiefly provided the instructive differentiation signal.

## Materials and methods

### Ethics Statement

The Hemocomponent Production and Validation Center (Centro per la Produzione e Validazione di Emoprodotti, CPVE) (Turin, Italy) Ethics Committee has waived the need for consent, due to the fact the blood donor material used was fully anonymized. The study did not involve human beings directly and, according to article 2 comma I, letter a) and article 6 of Italian Legislative Decree dated 24. 06. 2003, no. 211, and article 1, comma I of Italian Ministry of Health Decree dated 12. 05. 2006, did not require an opinion from the Ethical Committee.

### Cell lines

The pancreatic-cell line PT45 (kindly provided by Dr. M.F. Di Renzo, Department of Biomedical Sciences and Human Oncology, University of Turin, Italy) [19] was grown in RPMI 1640 medium supplemented with 10% fetal calf serum (FCS) (Merck Millipore, Billerica, MA). The cell line was routinely screened for mycoplasma contamination, using the Hoechst dye H33258 (Sigma Aldrich, St. Louis, MO, USA). Immortalized human pancreatic ductal epithelial cells HPDE6-E6E7 (H6c7), established after transduction of the HPV16-E6E7 genes into primary cultures of normal pancreatic duct epithelial cells, were generously provided by Dr. Ming-Sound Tsao, (Ontario Cancer Institute/Princess Margaret Hospital, University Health Network, Toronto, Canada) [20]. The cell line demonstrates a near-normal genotype and phenotype of pancreatic duct epithelial cells [21]. The H6c7 cells were grown in serum free Keratinocyte Basal Medium (KBM) fortified with growth factors, cytokines, and supplements (SingleQuots™ Kit) (Lonza Group Ltd, Basel, Switzerland).

In order to obtain serum-free conditioned medium (CM), PT45 and H6c7 cells were trypsinized, extensively washed with phosphate-buffered saline (PBS), and seeded at 3×10^5^/ml, in 5 ml of serum-free RPMI 1640 medium containing 0.25% fatty-acid-free bovine serum albumin fraction V (Sigma Aldrich) and KBM, respectively. After 48-hour incubation in a humidified atmosphere containing 5% CO_2_, CM was collected after centrifugation, and stored at -20°C until use.

### Human umbilical vein endothelial cells (HUVEC)

HUVEC were obtained from Lonza Group Ltd and propagated in EndoGRO medium (Merck Millipore).

### Isolation of circulating CD34^+^ progenitor cells

Low-density peripheral blood mononuclear cells (PBMC) were isolated from normal buffy coats received from the CPVE, by centrifugation at 250 *g* over Ficoll-Hypaque. CD34^+^ cells were purified using the CD34-isolation mini-MACS (magnetic cell sorting) kit (Miltenyi, Bergisch Gladbach, Germany) or the EasySep human CD34^+^ selection kit (StemCell Technologies, Vancouver, BC, Canada), following the manufacturers' directions. In both cases, the purity of the isolated CD34^+^ cells was verified by flow cytometry, and was in the 89%-98% range.

### RNA extraction and real time reverse transcription (RT)-PCR

RNA was extracted from PT45 cells, H6c7 cells, 19-day-cultured CD34^+^ cells in the absence or presence of PT45-CM, and from appropriate positive controls, comprising the colon carcinoma cell line DLD-1, the hepatocellular carcinoma cell line HepG2, bone marrow (BM) stroma, and HUVEC, using TRIzol Reagent (Invitrogen, Carlsbad, CA) and following the manufacturer’s instructions. To remove traces of genomic DNA, total RNA (3 μg) was treated with DNase I (Invitrogen) and reverse-transcribed to cDNA, using SuperScript II Reverse Transcriptase (Invitrogen). Lastly, cDNA specimens were diluted with 130 μl of H_2_O.

Real Time RT-PCR analysis was run on the iCycler iQ system (Bio-Rad, Hercules, CA) by the SYBR green I dye detection method. Amplification of *β-actin*, *VEGF-A, Angio-1, Angio-2, SDF-1A (CXCL12), VE-cadherin, von Willebrand factor* (*vWF*), *VEGF receptor (R)-1* (*flt-*1) and *VEGFR-2* (*flk* ***-*** *1* ***/*** *KDR*) transcripts was run in duplicate, in a PCR optical 96-well reaction plate (Bio-Rad); the PCR mixture in each well contained cDNA corresponding to 100 ng of total RNA, the relevant sequence-specific primers (150 nM for β-actin and 300 nM for the other cytokines) and 1X iQ SYBR Green Supermix (Bio-Rad). Table 1 lists primer sequences, designed using Beacon Designer 5 Software (Bio-Rad) to be cDNA specific, and to work under equivalent reaction conditions. Primers were synthesized by Invitrogen and reconstituted in nuclease-free water before use. Negative PCR controls without cDNA template, and a positive control sample with a known cycle threshold value (Ct), were included in each assay. Optimized thermal cycling conditions were as follow: 5 min at 95°C followed by 40 cycles of 15 s at 95°C and 1 min at 60°C (two-step PCR). Specificity of the PCR products was confirmed by the melting curve program at the end of the reaction (55°C to 95°C with a heating rate of 0.5°C/10 sec and continuous fluorescence measurements). PCR efficiency (*E*) was determined by the method described by Ramakers et al. [22] using the iCycler iQ software. For each sample, the Ct was acquired using the Fit Point Method [23]. Mean normalized gene expression (MNE) was calculated from the following formula: NE = *E*_*reference*_^Ct reference^/*E*_*target*_^Ct target^. The statistical significance of the difference in mRNA expression of the receptors examined, in PT45-CM treated- and untreated-CD34^+^ cells, was analyzed using the Relative Expression Software Tool (REST) for group-wise comparison, and statistical analysis of relative expression results in real-time PCR [24]. This software package calculates an expression ratio relative to the control group (untreated-CD34^+^ cells) normalized to a reference gene (*β-actin*). The mRNA expression data for *β-actin* showed no significant difference between untreated- and PT45-CM treated- CD34^+^ cells. The expression ratio (R) is: R = *E*_*target*_^ΔCt target (mean untreated-mean treated sample)^/*E*_*reference*_^ΔCt reference (mean untreated-mean treated sample)^.

**Table 1:** Primer sequences for housekeeping, cytokine and endothelial marker quantification by real time RT-PCR

**Primer set**	**GenBank**	**Primer sequence**	**RT-PCR**
	**Accession #**		**E (%)**
***β-actin***	NM_001101	FW -GCG AGA AGA TGA CCC AGA TC-	
		RW -GGA TAG CAC AGC CTG GAT AG-	100
***VEGF-A***	NM_001025366	FW -ATG AGG ACA CCG GCT CTG ACC A-	
		RW -AGG CTC CTG AAT CTT CCA GGC A-	94.5
***Angio-1***	NM_001146	FW -GAG GCA CGG AAG GAG TGT GCT G-	
		RW -CGG CGC TGA TTG CTG CAC CCT A-	92.0
***Angio-2***	NM_001147	FW -AAA AGC TGA CAC AGC CCT CCC A-	
		RW -ACT GCT GTG TTC TCT CCA GGC A-	86.0
***CXCL12***	NM_000609	FW -ATG CCC ATG CCG ATT CTT CG-	
		RW -GCC GGG CTA CAA TCT GAA GG-	100
***VE-cadherin***	NM_001795	FW -GGT CCC TGA ACG CCC TGG TAA-	
		RW -GGA GTG GAG TAT GGA GTT GGA GCA-	95.5
***von Willebrand Factor (vWF)***	NM_000552	FW -GCC TGC TTC TGC GAC ACC ATT G-	
		RW -GCC ACT CAC ACT CAT ACC CGT TCT-	96.2
***Flt1 (VEGF-R1)***	NM_002019	FW -CAC GCA GGA CCA GTT TGA TTG AG-	
		RW -GCT AGA GGA CTC CCG AGA TGT TG-	96.7
***Flk-1/KDR*** ***(VEGF-R2)***	NM_002253	FW -GCA GGG GAC AGA GGG ACT TG-	
		RW -GAG GCC ATC GCT GCA CTC A	95.3

### Evaluation of VEGF-A, CXCL12, Angio-1, and Angio-2

Secreted levels of VEGF-A, CXCL12, Angio-1, and Angio-2 were determined in cell-free CM from the pancreatic-carcinoma cell lines PT45 and H6c7, using commercially-available ELISA kits (R&D Systems, Minneapolis, MN, and RayBiotech, Norcross, GA, respectively) following the manufacturers’ directions. The minimum detectable doses were below 9 pg/ml for VEGF-A, below 18 pg/ml for CXCL12, below 30 pg/ml for Angio-1, and below 10 pg/ml for Angio-2.

### Cell migration assay

Cell migration assays were run in transwell plates (8 μm pore size, Costar, Cambridge, MA). Either CD34^+^ or HUVEC cell suspension (3×10^4^ cells/ml) was added to the upper compartment of the transwell chamber. 30% of PT45- or of H6c7-CM, as such or pre-treated with neutralizing polyclonal goat antibodies against VEGF-A (Chemicon International, Temecula, CA) and Angio-1 (Sigma Aldrich), or with appropriate irrelevant antibody as control, was added to the lower well of the transwell chamber as chemoattractant. In selected experiments, CD34^+^ cells were also pretreated with an anti-human VEGFR-1/Flt-1 polyclonal goat antibody blocking receptor-ligand interaction (25 μg/ml) (R&D Systems). The chambers were incubated for 24 h at 37°C, 5% CO_2_. After removing the cells from the upper side of the membrane, with a cotton swab, migrated cells were fixed and stained with crystal violet dye. Stained cells were visualized and quantified by counting cells in three high-power fields (100X). Data are shown as means ± SE from three/six separate series of assays.

### Proliferation assay

CD34^+^ cells were seeded in triplicate at a concentration of 5×10^4^ cells/well in 96-well plates, in RPMI 1640 medium containing 10% FCS, in the absence or presence of 30% of either PT45-CM or H6c7-CM. 50 ng/ml of human recombinant Stem Cell Factor (SCF) and Fms-related tyrosine kinase (FLT)-3 Ligand (PeproTech, Rocky Hill, NJ) was used as control, to activate stem-cell and immature-progenitor-cell cycling. To determine DNA synthesis, after 20 hr incubation, cells were pulsed with triziated-Thymidine (^3^H)-TdR (Perkin Elmer, 1 μCi/well) for a further 4 hr. Cellular DNA was collected on glass fiber filters, and ^3^H-TdR incorporation was measured in a β-counter. Results are expressed as median cpm (count per minute) (range) of two separate experiments.

### Differentiation of CD34^+^ cells

To determine the effect of tumor-derived soluble factors on CD34^+^ cell differentiation, normal peripheral blood CD34^+^ progenitor cells, isolated immunomagnetically as described above, were resuspended in Iscove medium (Merck Millipore) containing 20% FCS, 10 ng/ml recombinant Interleukin (IL)-3, 10 ng/ml SCF, and 50 ng/ml Granulocyte-Macrophage Colony Stimulating Factor (GM-CSF) (PeproTech); they were then seeded in wells of 6-well plates coated with fibronectin, in the absence or presence of 30% PT45-CM. Cells were allowed to incubate for 15-19 days at 37°C in a 5% CO_2_ atmosphere.

### Evaluation of acetylated low-density lipoprotein uptake and of binding to *Ulex europaeus* lectin, by laser scanning confocal microscopy (LSCM)

To measure the uptake of acetylated low-density lipoprotein, and adherence to *Ulex europaeus* lectin, CD34^+^ cells were grown in thick-glass-base dishes (Asahi Techno Glass Co., Singapore). After 15-19 days’ culture in the presence of IL-3, SCF, and GM-CSF, with or without 30% of PT45-CM, cells were incubated for 1 h at 37°C in RPMI + 10% FCS containing 10 μg/ml 1,1-dioctadecyl-3,3,3,3-tetramethylindocarbocyanine-labeled acetylated low-density lipoprotein (Dil-AcLDL; Molecular Probes, Eugene, USA) and fixed with 2% paraformaldehyde for 10 min. After washing, fluorescein isothiocyanate (FITC)-conjugated *Ulex europaeus* lectin (Sigma Aldrich) was added at a concentration of 10 μg/ml in PBS, and cells were incubated for 1 h at room temperature in the dark. HUVEC were used as positive control. Direct fluorescence staining was analyzed by LSCM (Zeiss LSM 510; Carl Zeiss SpA, Arese, Milan, Italy) equipped with a Zeiss inverted microscope with a “plan neofluar” lens. Exciting light intensity, black level, and photomultiplier gain were adjusted on control specimens; the same settings were used to scan experimental slides. The laser band of excitation was 488/543 nm; the emission was set with a 560 nm long-pass filter for Dil-AcLDL (red) and a 505-530 nm band pass for FITC-conjugated *Ulex europaeus* lectin (green). The fluorescent images were processed using LSCM 510 Image Examiner software from Zeiss.

### Flow cytometry analysis

Expression of endothelial cell markers CD146 and CD105 was analyzed on untreated and on PT45-CM-treated CD34^+^ cells, after 19 days’ culture, in the presence of GM-CSF, IL-3 and SCF. The cells were stained with FITC-conjugated CD146 (Chemicon International) and CD105 (Millipore) mouse monoclonal antibodies for 30 min at 4°C. After washing, cells were analyzed by flow cytometry in a Coulter Epics IV Cytometer (Beckman Coulter, Inc., Fullerton, CA). Results are expressed as percentages of positive cells. Statistical analyses were based on at least 30,000 events gated on the population of interest.

### Immunohistochemistry

PT45 cells, and 19-day cultured CD34^+^ cells in the presence of IL-3, SCF and GM-CSF, with or without 30% of PT45-CM, were harvested, cytospinned and fixed in acetone. After 5 min exposure to 0.3% solution of H_2_O_2,_ to inhibit endogenous peroxidase activity, cytospins were treated for 1 h with a blocking buffer (10% normal mouse/rabbit/goat serum in PBS) to prevent nonspecific staining, incubated overnight at 4°C with the primary antibodies listed in Table 2, and then washed twice with PBS for 5 min each time. For negative controls, the primary antibodies were replaced with appropriate non-immune sera. The slides were then treated with appropriate biotinylated secondary antibodies, followed by incubation with avidin-biotin peroxidase complex solution, for 30 min, and diaminobenzidine tetrahydrochloride as chromogen (Dako LSAB® kit, Dako, Glostrup, Denmark). The slides were finally counterstained with Mayer's hematoxylin for 5 seconds, dehydrated, and mounted in Clarion (Biomeda, Foster City, California, USA).

**Table 2:** Antibodies and immunohistochemical procedures

**Reactivity**	**Antibody**	**Source**	**Antibody**
	**Species**		**Dilution**
**VEGF-A**	Rabbit	Santa Cruz Biotech., Santa Cruz, CA	1:20
**Angio-1/4**	Goat	Santa Cruz Biotech	1:200
**Angio-2**	Goat	Santa Cruz Biotech	1:200
**CXCL12**	Rabbit	Santa Cruz Biotech	1:30
**VE-cadherin**	Mouse	Santa Cruz Biotech	1:100
**vWF related antigen**	Rabbit	Shandon Immunon, Pittsburgh, PA	1:20

### *In vitro* angiogenesis assays

Matrigel basement membrane matrix (Becton Dickinson, Franklin Lakes, NJ USA) was added (100 μl) to a 96-well plate and allowed to polymerize. HUVEC or CD34^+^ cells, cultured for 19 days in IL-3, SCF and GM-CSF, in the presence or absence of 30% of PT45-CM, were resuspended in Medium 199 (Millipore) and 0.5% FCS (2×10^4^ cells/1 ml) in the presence of 30% of PT45-CM, pre-treated or not with neutralizing antibodies against VEGF or Angio-1, or with the appropriate irrelevant antibody; they were then plated (100 μl) on the top of the solidified matrix solution and incubated at 37 C for 24 hr. Tubule formation was visualized using an inverted-light microscope with a 20X objective (Zeiss), and images of ten random fields were captured using a digital camera. Each cell within the optical field was counted, and this number referred to as the "total number of cells per field". The angiogenic potential was quantified from the following formula:

Angiogenic score = [(number of sprouting cells x 1] + number of connected cells x 2 + number of polygons x 3)]/ Total number of cells per field. The final individual score derived from the formula was then taken as one value in a total of ten, with median [range] represented against other treatment conditions.

### Statistical analysis

To assess statistically-significant differences between datasets, Student's *t* tests for paired samples, or Mann-Whitney Rank Sum tests, were performed using SigmaStat software (Jandel Scientific, San Rafael, CA).

## Results

### Expression of angiogenic factor mRNA in PT45 and in H6c7 cells by real time RT-PCR

Steady-state angiogenic factor mRNA levels were quantitatively assessed in PT45 and H6c7 cell lines, using real-time RT-PCR, and normalized gene expressions were compared (Table 3). Both cell lines expressed similar levels of *VEGF* mRNA. Conversely, the *Angio-1* transcript was expressed at a markedly lower level in H6c7 cells than in PT45 cells (fourteen times lower). Both cell lines were found to be negative for *Angio-2* mRNA. The *CXCL12* message was expressed at a very low level in PT45 cells and not at all in H6c7 cells.

**Table 3:** Levels of angiogenic factor mRNA in PT45 and H6c7 cells

**Gene**	**mRNA levels (relative to β-actin)**	
	**PT45 cells H6c7 cells**	
***VEGF-A***	0,016821^a^	0,016270
***Angio-1***	0,000083	0,000006
***Angio-2***	nd^b^	nd
***CXCL12***	0,000001	nd

^a^Mean normalized gene expression (MNE).

^b^not detectable.

### Expression of VEGF-A, Angio-1, Angio-2 and CXCL12 proteins by PT45 and by H6c7 cells

**Figure 1:**
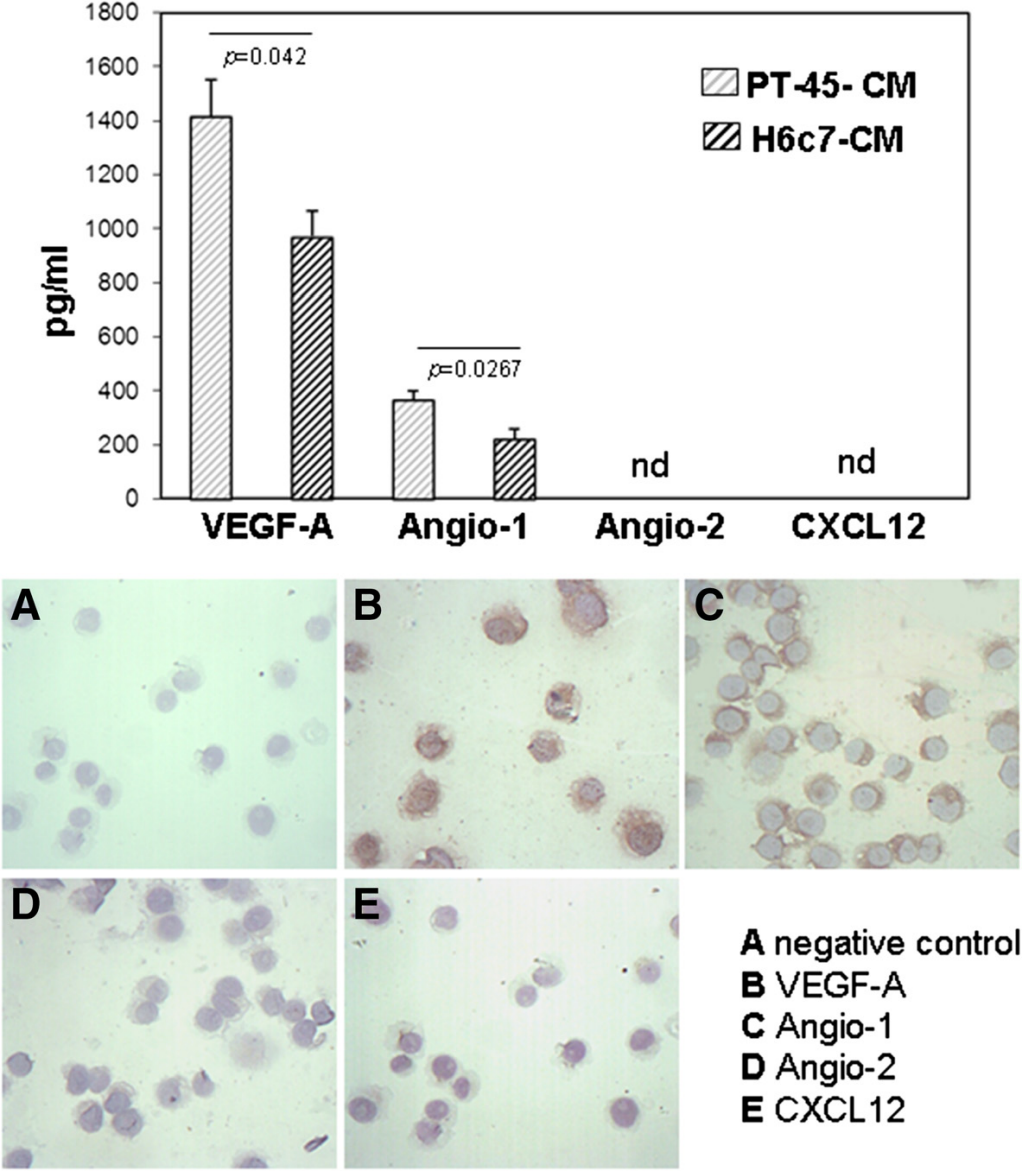
**Concentrations of VEGF-A, Angio-1, Angio-2 and CXCL12 in CM derived from PT45 and H6c7 cell lines measured by ELISA.** Values are means ± SE of angiogenic factors detected in duplicate CM samples, in five separate experiments for PT45-CM, and in three for H6c7-CM (upper panel). Detection of VEGF-A, Angio-1, Angio-2 and CXCL12 in pancreatic carcinoma cells PT45, determined by immunohistochemistry. A representative negative control is also shown (original magnification 250X) (lower panel).

The release of VEGF-A, Angio-1, Angio-2, and CXCL12 proteins was evaluated in PT45-CM and in H6c7-CM by ELISA. As shown in Figure 1 (upper panel), significantly higher levels of VEGF-A and Angio-1 were detected in PT45-CM than in H6c7-CM (mean ± SE: 1413.8 ± 311.6 pg/ml vs. 971.3 ± 186.5 pg/ml, p = 0,042; mean ± SE: 362.9 ± 37.9 pg/ml vs. 224.4 ± 33.14 pg/ml, p = 0,0267, respectively). Consistently with mRNA expression, Angio-2 and CXCL12 levels were undetectable by ELISA in either cell supernatants. Immunohistochemical analysis of these molecules, run on PT45 cell cytospins using specific antibodies, detected intense staining for VEGF-A and Angio-1 in the cytoplasm of the majority of PT45 cells, while Angio-2 and CXCL12 expression was negligible (Figure 1, lower panel), consistently with the ELISA results. The combined expression of VEGF-A and Angio-1 suggests that the poorly-differentiated PT45 cells possess higher vascular remodeling capacity than do H6c7 cells.

### Do PT45-CM and H6c7-CM chemoattract circulating CD34^+^?

**Figure 2:**
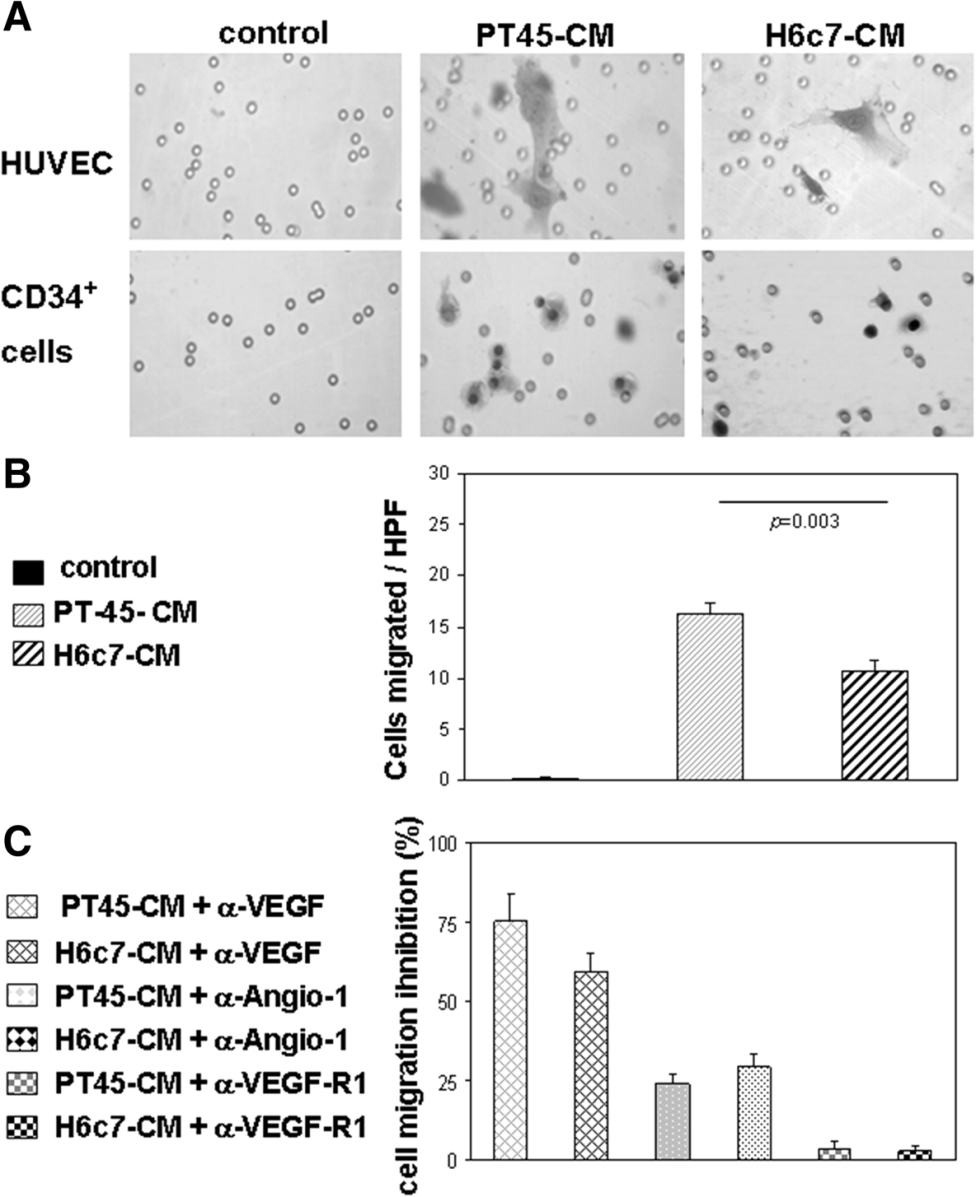
**Chemo-attraction of CD34**^**+ **^**cells by PT45-CM and H6c7-CM.** PB CD34^+^ cells and HUVEC were seeded into the upper compartment of a chemotaxis chamber. PT45- or H6c7-CM (30%) was placed in the lower compartment, as such or pre-treated with neutralizing anti-VEGF-A and anti-Angio-1 antibodies. In selected experiments, CD34^+^ cells were pre-treated with a neutralizing anti-VEGF-R1 antibody before exposure to PT45- or to H6c7-CM. After 24 hr incubation, cells in the upper compartment were removed, and the number of CD34^+^ or HUVEC cells that had migrated from the upper compartment through the filter into the lower compartment was determined, by counting the cells in three random high-power fields. **(A)** Representative images of HUVEC and CD34^+^ cells migrating in response to medium alone (control), to PT45-CM or to H6c7-CM, in a transwell migration assay. **(B)** Number of cells migrating in a transwell migration assay, in the absence or presence of PT45-CM or H6c7-CM. Results are mean values ± SE of six separate series of studies for PT45-CM and of three series for H6c7-CM. **(C)** Inhibitory effect of a neutralizing anti-VEGF-R1 antibody on CD34^+^ cell chemotaxis; values are expressed as percentage inhibition of CD34^+^ cell migration in the presence of PT45-CM/H6c7-CM versus medium alone (control). Data shown are means ± SE from three separate series.

To test this hypothesis, since production of endothelial chemotactic factors, such as VEGF-A and Angio-1 [25], was detected in PT45-CM and in H6c7-CM, the supernatants were assessed for *in vitro* chemotaxis of circulating CD34^+^ cells, which are known to express the relative receptors [26]. HUVEC were used as positive controls. As shown in Figure 2, both HUVEC and CD34^+^ cells demonstrated a significant chemotactic response, both to PT45-CM and to HPDE-CM, although in the latter case to a lesser extent (CD34^+^ cells migrated/HPF: PT45-CM, 16.333 ± 3.082 *vs*. H6c7-CM, 9.667 ± 3.41, *p* = 0.003). Since factors secreted by PT45 and H6c7 cells are also likely to have an effect on cell proliferation, to ensure that the results are a true measure of cell migration, CD34^+^ cells were cultured for 24 hr in the presence or absence of PT45-CM or H6c7-CM, and DNA-synthetic activity was assessed in terms of ^3^H-TdR uptake. No significant difference in DNA synthesis (p >0.05) was observed between untreated and CM-treated CD34^+^ cells [median cpm (range): control 438 (334-527); PT45-CM 395 (296-478); H6c7-CM 380 (297-502)]. In the presence of the mitogenic factors SCF and FLT-3, the DNA-synthetic activity of CD34^+^ cells slightly increased over control values, but not to a statistically-significant extent (median cpm (range): 531 (344-727) vs. 438 (334-527), p = 0,400). To determine the factor responsible for chemoattraction of CD34^+^ cells, the supernatants were pre-treated with isotype-control or with neutralizing VEGF-A and Angio-1 antibodies. Treatment with antibodies neutralizing Angio-1 activity only partially reduced the CD34^+^ migration induced by either CM (% CD34^+^ cells migration inhibition: PT45-CM, 24.167 ± 8.503%; H6c7-CM, 29.134 ± 13.429%), whereas when the neutralizing VEGF-A antibody was added, migration was almost entirely inhibited (% CD34^+^ cells migration inhibition: PT45-CM, 75.167 ± 25.87%; H6c7-CM, 59.476 ± 17.359%). By contrast, exposure to a neutralizing VEGF-R1 polyclonal antibody did not significantly decrease CD34^+^ recruitment (% CD34^+^ cells migration inhibition: PT45-CM, 3.594 ± 5.843%; H6c7-CM, 2.891 ± 2.3%). Inclusion of the isotype control antibodies had no effect (data not shown). These results demonstrate that, both in normal and in neoplastic conditions, the chemoattractant activity exerted by H6c7 cells on CD34^+^ cells is mainly due to VEGF, and that the responding cells are included in the CD34^+^/VEGF-R2^+^ progenitor cell subset.

### Morphological changes of CD34^+^ cells cultured in the presence of PT45-CM

**Figure 3:**
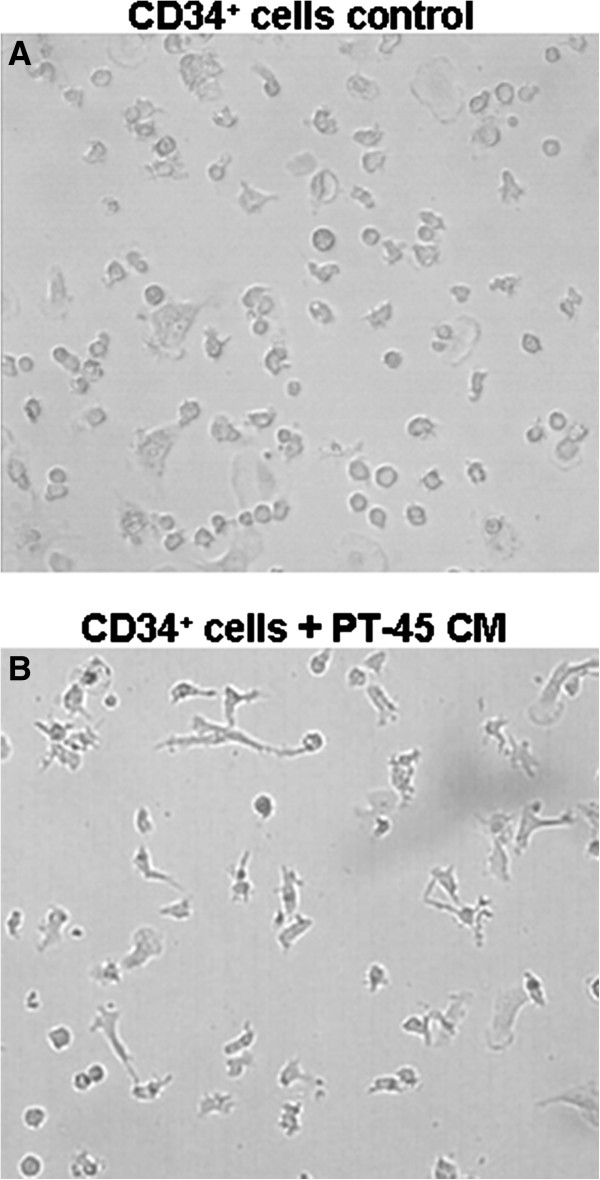
**Altered morphology during differentiation of CD34**^**+ **^**cells in the presence of PT45-CM.** Circulating CD34^+^ cells were cultured for 19 days in the presence of GM-CSF, IL-3 and SCF, either in the absence or in the presence of PT45-CM. Shown are representative examples of cells cultured in GM-CSF, IL-3 and SCF alone **(A)**; as panel A, but including PT45-CM **(B)**.

The finding that the CD34^+^ cell subset is recruited by PT45-CM, along with the higher tumor-derived levels of VEGF-A and Angio-1 that play prominent roles in the vascularization process, prompted us to investigate whether tumor cells might cause differentiation of these CD34^+^ progenitor cells along the myeloid lineage to be switched toward the endothelial cell lineage. This was investigated by culturing purified normal PB CD34^+^ cells for 19 days, under experimental conditions recognized as supporting their differentiation into myeloid cells (IL-3, SCF and GM-CSF) [27], but in the presence or absence of PT45-CM. The hypothesis that tumor-CM was actively altering the differentiation of CD34^+^ cells was initially supported by morphological evaluation of the resulting cells: as shown in Figure 3, in control cultures, the cells that developed from the CD34^+^ cells tended to be rounded. By contrast, in cultures with tumor-CM, a number of cells (median 7%, range 4-18) appeared more elongated, spindle-shaped, with areas within the culture containing cells organized in long head-to-tail configurations.

### Characterization of CD34^+^ cultured in the presence of PT45-CM

**Figure 4:**
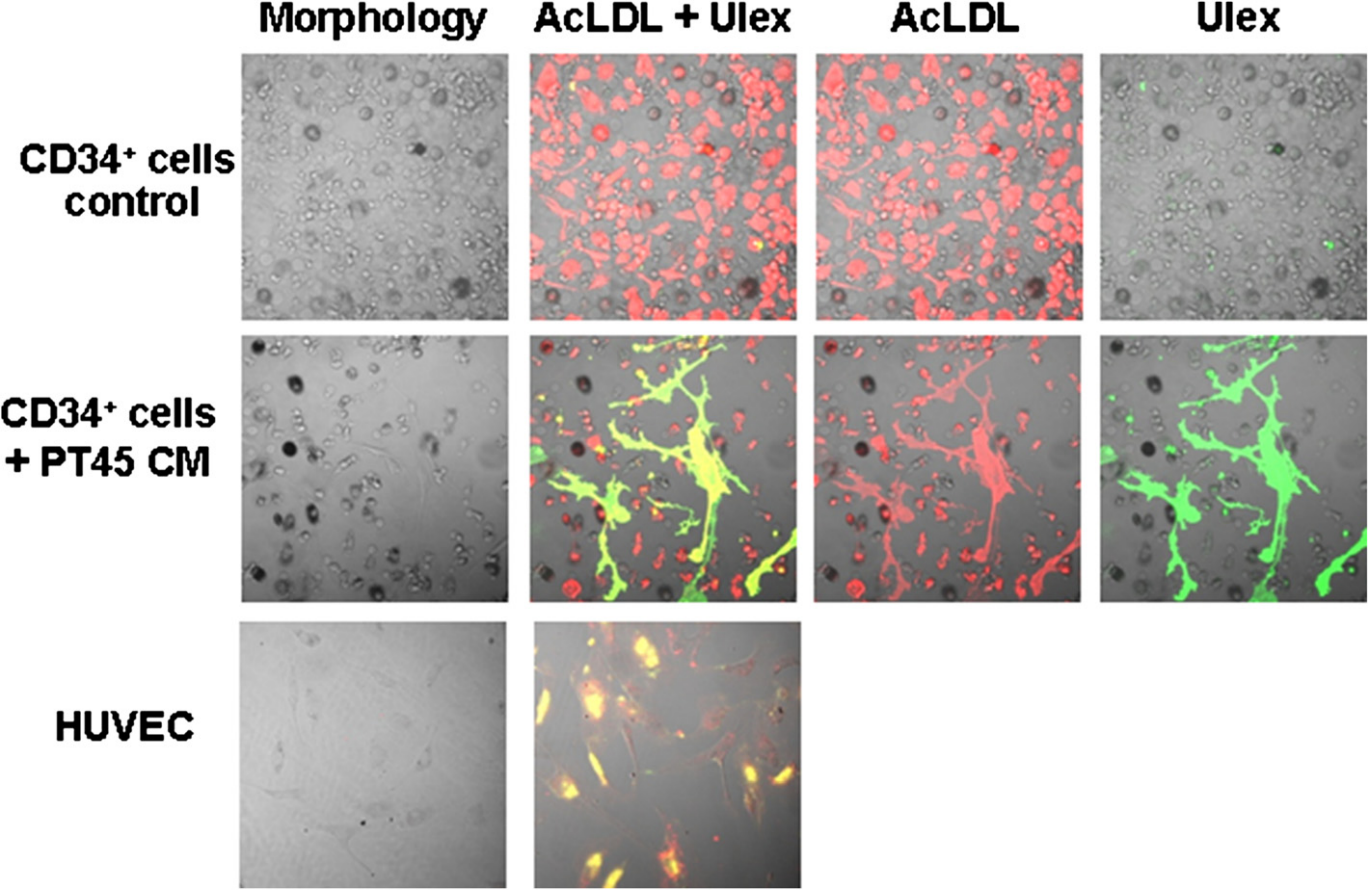
**Fluorescence staining of cultured CD34**^**+ **^**cells with Dil-AcLDL and *Ulex europeus *lectin under an LSCM.** Circulating CD34^+^ cells were cultured for 19 days in the presence of GM-CSF, IL-3 and SCF, either without or with PT45-CM. The laser band of excitation was 488/543 nm; the emission was set with a 560 nm long-pass filter for Dil-AcLDL (red) and a 505–530 nm band pass for FITC conjugated *Ulex europeus* lectin (green). Photographs are from one representative experiment. The lens used was 20×/0.5 (0.849 × 0.849 mm^2^ image dimension). The fluorescent images were processed using an LSCM 510 Image Examiner Program from Zeiss: the yellow merged image represents the result of double-fluorescence labeling.

Tests were then run to determine whether or not endothelial cells develop in cultures containing PT45-CM, by means of uptake of Dil-AcLDL and *Ulex europeus* lectin binding; these activities are characteristic of endothelial cells [28,29]. We assayed these capacities in the 19-day cultures, by both double immunofluorescence staining and LSCM. We identified and quantified a small proportion (9-15%) of cells showing double-positive staining for Dil-AcLDL uptake and binding of *Ulex europeus* lectin (Figure 4). These results indicate that this endothelial-like fraction originates in the hematopoietic stem-cell-containing CD34^+^ cell population only in the presence of tumor-CM.

### Quantitative analysis of mRNA expression of VE-cadherin, vWF, VEGF-R1 and VEGF-R2

**Figure 5:**
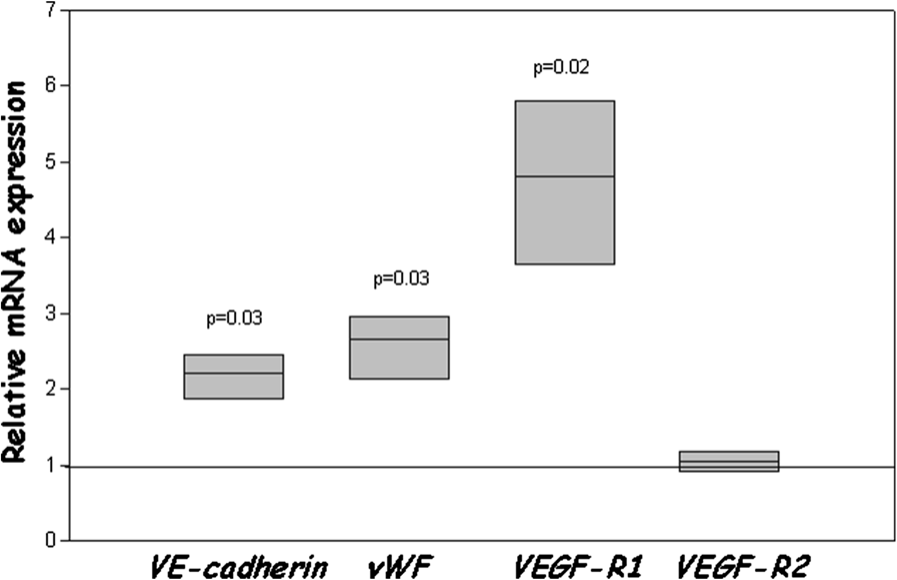
**Levels of *VE-cadherin, vWF, VEGF-R1 *and *VEGF-R2 *mRNA in CD34**^**+ **^**cultured with or without PT45-CM.** Cells were cultured for 19 days under conditions supporting their differentiation toward myeloid lineage cells, in the presence or absence of PT45-CM. Results were assessed by real time RT-PCR, and normalized to β*-actin* mRNA levels. Normalized gene expression values relative to that of control CD34^+^ cells (cultured in the absence of PT45-CM, arbitrarily set to 1) are shown.

Using real timeRT-PCR, the expression of genes correlated with endothelial cell lineage differentiation was then evaluated, in CD34^+^ cells cultured for 19 days in the presence of IL-3, GM-CSF and SCF, with or without 30% PT45-CM. The results, shown in Figure 5, demonstrate that *VE-cadherin*, *vWF* and in particular the *VEGF-R1* (*flt-1*) transcripts were more strongly expressed in CD34^+^ cells cultivated in the presence of PT45-CM, versus controls (*p* = 0.03, *p* = 0.03 and *p* = 0.02, respectively). Conversely, the expression of *VEGF-R2* [*KDR or flk-1*] was not significantly modulated in the presence of PT45-CM.

### Expression of endothelial-cell markers on CD34^+^ cells cultured in the presence of PT45-CM

**Figure 6:**
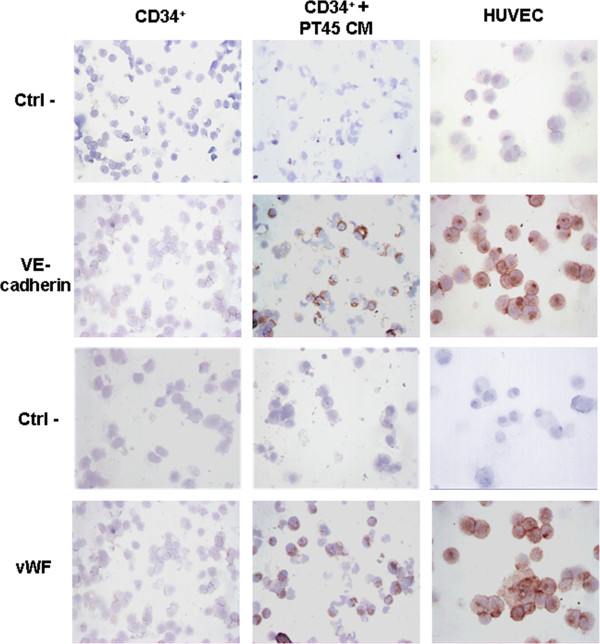
**Detection by immunohistochemistry of VE-cadherin and vWF in CD34+ cells cultured with or without PT45-CM.** Cells were cultured for 19 days under conditions supporting their differentiation toward myeloid lineage cells, in the presence or absence of PT45-CM. Specimens are representative examples of nine different experiments. Representative negative controls are also shown (original magnification 250X).

Cells derived from CD34^+^ cells cultured for 19 days with or without PT45-CM, under conditions that support myeloid differentiation, were analyzed for endothelial-cell-marker expression, by flow cytometry (CD146 and CD105) and by immunohistochemistry (vWF and VE-cadherin). As shown in Table 4, in the absence of tumor-CM, a small proportion of cells were positive for CD146 and CD105. However, when PT45-CM was also added to the CD34^+^ cell cultures, a significantly greater number of cells expressed CD146 and CD105 (p = 0.003 and p = 0.006, respectively,). As shown in Figure 6, exposure to tumor-derived angiogenic factors promoted the expression of VE-cadherin and vWF, both of which are endothelial-specific markers, in a subset of cultured CD34^+^ cells (24 ± 8% and 23 ± 10%, respectively).

**Table 4:** Flow cytofluorimetric analysis of endothelial marker CD146 and CD105

**Colture conditions of CD34**^**+ **^**cells**	**% positive cells (mean ± SE)**			
	**CD146 *p***^***a ***^**CD105 *p***			
**GM-CSF, IL-3, SCF**	12.14 ± 5.9		18.14 ± 3	
**GM-CSF, IL-3, SCF + 30% PT45-CM**	21.84 ± 7.7	0.003	28.1 ± 2.1	0.006

^a^*vs* CD34^+^ cultured in the presence of IL-3, GM-CSF and SCF.

### *In vitro* angiogenesis assays

**Figure 7:**
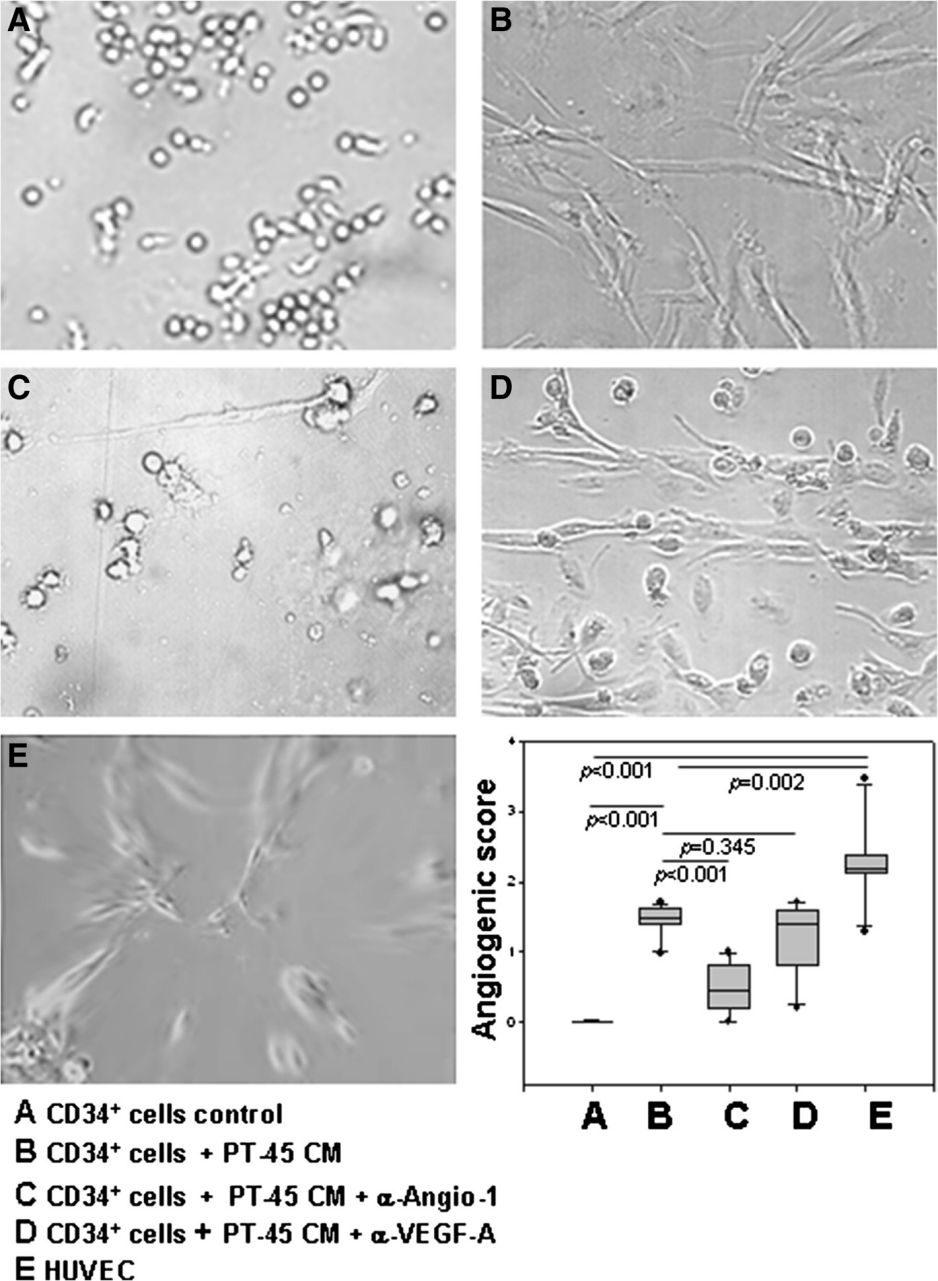
**In vitro angiogenic potential of PT45-CM treated CD34**^**+ **^**cells.** CD34^+^ cells were grown on Matrigel for 24 h, after being cultured for 19 days under conditions supporting their differentiation toward myeloid lineage cells, in the absence **(A)** or presence **(B)** of PT45-CM, pre-treated or not with a neutralizing anti-Angio-1 antibody **(C)** and with a neutralizing anti-VEGF-A **(D)** antibody. HUVEC **(E)** were used as positive control. The figure is representative of three experiments done in duplicate; the results are presented as median (range) values of the angiogenic numerical score, derived from the formula reported in the Materials and Methods section.

Using an *in vitro* angiogenesis assay on Matrigel, the angiogenic potential of HUVEC was compared to that of untreated or PT45-CM-treated CD34^+^ cells, cultured for 19 days in the presence of IL-3, GM-CSF and SCF. Figure 7 A-E shows a representative tubulogenesis assay. On Matrigel, HUVEC formed a well-organized network of cordlike structures, with a median (range) angiogenic score of 2.19 (1.29-3.46) (E); conversely, in CD34^+^ cell control cultures, no endothelial organization was observed [median (range) angiogenic score: 0 (0-0.02)] (A). PT45-CM exposed CD34^+^ cells exhibited extended protrusions, connections, and alignments, but tube formation was less pronounced [median (range) angiogenic score of 1.47 (0.98-1.70), *p* = 0,002 vs. HUVEC and *p* < 0.001 vs. CD34^+^ control cells] (B). The formation of capillary-like structures by PT45-CM treated CD34^+^ cells was significantly reduced by the use of a neutralizing anti-Angio-1 antibody [median (range) angiogenic score of 0.45 (0-1), *p* < 0.001 vs. PT45-CM-exposed CD34^+^ cells] (C), but not when PT45-CM was pre-treated with a neutralizing anti-VEGF-A antibody [median (range) angiogenic score of 1.39 (0.20-1.72), *p* = 0.345 vs. PT45-CM exposed CD34^+^ cells] (D). Non-immune goat IgG did not affect cell organization (not shown).

## Discussion

Expression of the CD34 surface antigen characterizes a heterogeneous population of BM-derived cells, including hematopoietic progenitor cells (HPC) and EPC [30], which can differentiate into mature endothelial cells when recruited to angiogenic sites. This in situ development of vessels, known as vasculogenesis, is prominent during embryonic development. However, although tissue remodeling and regeneration in postnatal life are mainly due to angiogenesis, involving preexisting vessels, it has recently been found that also vasculogenesis can occur in the adult, particularly during wound healing and tumor vascularization [31,32]. In cancer patients, including those with advanced pancreatic carcinoma, EPC are numerous compared to healthy controls; EPC numbers also correlate positively with circulating VEGF-A levels, and inversely with disease stage and prognosis [17,33,34]. These findings combine to suggest that tumor microenvironmental conditions may foster the recruitment, mobilization, and differentiation of EPC [35]. Both tumor cells themselves, and tumor-educated immune cells, locally secrete a broad spectrum of biologically-active factors that facilitate cancer growth.

In a previous study that examined an array of tumor-derived cytokines affecting the immune response, we found that the poorly-differentiated pancreatic carcinoma cell line PT45 produced high levels of VEGF-A [10]. The present study shows that, among other relevant angiogenic factors including the angiopoietins and CXCL12, PT45 cells also express *Angio-1* mRNA and protein. However, these vasculogenic cytokines are not tumor-exclusive products, but are thought to be part of the complex milieu involved in maintaining the vasculature in normal tissues: they are also expressed, albeit to a lesser extent, in the near-normal HPDE H6c7 cells. VEGF-A, released by both malignant (PT45) and normal (H6c7) cells, appears to play a major role, with regard to cell targets (CD34^+^ VEGF-R2^+^) and functional chemotactic activity.

Unlike the physiological restoration of vascular perfusion to damaged tissues, blood vessel development in solid tumors is not closely controlled, but continues relentlessly. Tumors exploit a number of strategies to achieve vascularization (alteration of local normal equilibrium of anti and pro-angiogenic stimuli, co-optation of pre-existing vessels, stimulation of intussusceptive microvascular growth, post-natal vasculogenesis, and/or vasculogenic mimicry) [36], that have profound consequences on tumor growth, metastasis, and response to therapy. It thus appears possible that pancreatic tumor cells, influenced by their microenvironment, might also exploit abnormal levels of normally-beneficial angiogenic factors, such as VEGF and Angio-1, as one strategy to achieve vascularization.

The present study shows that, presumably thanks to an abnormally elevated gradient of angiogenic growth factors*,* medium conditioned by PT45 pancreatic carcinoma cells affects differentiation of a subset of CD34^+^ cells into endothelial cells, producing conditions that support their differentiation toward myeloid lineage cells [27]. It is well-known that the high levels of VEGF produced by tumors can mobilize BM-derived stem cells in the peripheral circulation, and enhance their recruitment to the tumor vasculature [37,38]. Moreover, Angio-1 modulates several aspects of angiogenesis, including remodeling and vascular permeability, and, in conjunction with VEGF, it also plays an essential role in promoting the survival of endothelial and hematopoietic progenitor cells [39]. We show here the existence of a subpopulation of CD34^+^ cells (10-15%) that, cultured under pro-myeloid differentiative conditions in the presence of PT45-CM, acquire what are often referred to as “endothelial cell lineage markers”, namely spindle shape, incorporation of Dil-AClDl, and binding to *Ulex-lectin*[28,29]. This suggests that, among CD34^+^ cells, endothelial stem cells/progenitor cells may exist. In general, early EPC in the BM, or immediately after their migration into the systemic circulation, are positive for CD133/CD34/VEGF-R2, whereas circulating EPC are positive for CD34/VEGF-R2/CD31 [40]. This indicates that at least two types of EPC are simultaneously present in the peripheral blood, and that the cells can change their progenitor properties while in the circulation. However, since purified CD34^+^ cells were concomitantly cultured in the presence of IL-3, SCF and GM-CSF (all of which are survival and/or myeloid differentiation factors) it cannot be ruled out that these cells might originate from the monocyte/macrophage lineage, as other reports have suggested [41,42]. Although it has been suggested that myeloid cells may express specific endothelial markers (such as CD34, CD31 and VEGF-R2), and that they may localize adjacent to blood vessels, and differentiate into endothelial cells [43,44], this hypothesis remains controversial; further research will be needed to clarify the situation.

In the present study, mRNA of both *Flt-1* and *KDR* (genes encoding, respectively, for VEGF-R1 and VEGF-R2) were detected in untreated CD34^+^ cells. After 19 days’ culture in the presence of IL-3, SCF and GM-CSF, with 30% PT45-CM, the *Flt-1* transcript significantly increased, while *KDR* was unchanged. In a study on angiogenic-defective tumor-resistant Id-mutant mice, the restoration of tumor angiogenesis, after transplantation of wild-type BM or VEGF-mobilized stem cells, was associated not only with the uptake of BM-derived VEGF-R2^+^-endothelial precursor cells in the blood vessels, but also with the incorporation of BM-derived myeloid cells into perivascular sites of the tumor microenvironment, these cells being characterized by the expression of VEGF-R1 [45]. It has been suggested that VEGF-R1^+^ cells may differentiate into pericyte-like cells and play a role similar to pericytes in the tumor microenvironment [46].

It may, thus, be speculated that VEGF release by PT45 cells might concomitantly promote CD34^+^ VEGF-R2^+^ cell differentiation along the endothelial lineage, this differentiation being characterized by the expression of vWF and VE-cadherin [47]. VEGF release might also induce the appearance of pericyte-like CD34^+^ VEGF-R1^+^ cells, which play mutually-supporting roles in tumor-vessel neoformation and stabilization, respectively. This hypothesis is supported by the finding that, after exposure for 19 days to PT45-CM in the presence of IL-3, SCF and GM-CSF, some CD34^+^ cells began to express specific endothelial markers, namely CD146, CD105, VE-cadherin and vWF, and a subset of cells started to express *VEGF-R1* RNAm. Interestingly, in human tissues from patients with malignancies, VEGF-R1^+^ cell clusters have been observed in both primary tumor and metastatic tissue; increased numbers of VEGF-R1^+^ clusters have also been found at common sites of metastasis even before tumor spread, suggesting this tissue might be a potential future site for metastasis [48]. PT45 cells also produce and release Angio-1, the natural ligand for tunica internal endothelial cell kinase (Tie)-2 receptor, [49] which, in conjunction with VEGF, modulates some aspects of angiogenesis, including remodeling and vascular permeability [50].

In our study, a subset of CD34^+^ cells, cultured under conditions that support their differentiation toward myeloid lineage cells, acquired expression of Tie-2 (data not shown) whether or not the culture medium comprised PT45-CM. Interest in cells of the myeloid lineage in regard to tumor angiogenesis has recently been revived, thanks to the observation that tumor-infiltrating Tie-2-expressing monocytes (TEM) convey pro-angiogenic programs in mouse models. TEM preferentially reside around newly formed tumor blood vessels in viable tumor areas [51,52]. It may thus be speculated that tumor-derived Angio-1 not only has a direct effect on endothelial cell behavior during vascular remodeling, but may also play a role in maintaining interactions between endothelial cells and support cells.

Since completing the tumor vasculogenesis process involves aligning endothelial cells and forming a three-dimensional network of tubes incorporating a functional microvasculature, it was also evaluated whether CD34^+^ cultured in Matrigel, in the presence of IL-3, SCF and GM-CSF with or without PT45-CM, could organize into a capillary-like structure. CD34^+^ cells, pre-exposed to PT45-CM, appeared markedly flattened; they migrated throughout the Matrigel surface and formed a network of interconnecting cells. Conversely, non-pre-exposed CD34^+^ cells were small, round in shape, and did not spread. The fact that the introduction of an anti-Angio-1 blocking antibody caused the CD34^+^ cells to remain grouped suggests that Angio-1, released by PT45 cells, favors the formation of capillary-like structures, while VEGF-A apparently has no effect on endothelial organization.

Particularly controversial points in the relevance of vasculogenesis to cancer progression are whether EPC are mobilized in response to cytokine release, either by tumor cells or by damaged tissues/host immune cells, and whether the role of EPC in angiogenesis is merely regulatory, or whether they form part of the new tumor vasculature [53]. Our study shows that circulating EPC may be chemo-attracted, and develop into endothelial-like cells that can form cord-like structures in vitro, under the direction of factors released by pancreatic tumor cells. This might clarify the processes whereby pancreatic-tumor-mobilized EPC contribute to vessel formation, and in any case adds a possible explanation for the observation of increased circulating EPC in pancreatic carcinoma [10].

Blocking recruitment of EPC may be essential in order to overcome their negative consequences in terms of patient survival. Preclinical and clinical evidence suggests an association between treatment with certain drugs (including 5-fluouracil, the drug most commonly used to treat advanced/metastatic pancreatic cancer) and increased levels of BM-derived EPC and VEGF-R1^+^ cells; these cells stimulate tumor progression and metastasis [54-56]. Other conventional drugs, such as gemcitabine and cisplatinum, show no similar association [57].

Numerous clinical trials employing antiangiogenic drugs for the treatment of pancreatic cancer have thus far failed to produce improvements in treatment [58]. One of the possible mechanisms underlying this general resistance is poor tissue perfusion, which limits drug delivery. Olive *et al*. provided evidence that, in *KRAS* and *p53* mutant pancreatic adenocarcinoma xenografts in a gemcitabine-resistant mouse model, chemotherapy does not reach tumor cells due to poor tumor vascularization [59]. Approaches involving antiangiogenic therapy are thus counterintuitive, given that destruction of the tumor vasculature might be expected to impair drug delivery.

Pancreatic carcinoma is generally surrounded by dense fibro-inflammatory tissue that creates unusually high interstitial fluid pressure (IFP); this high pressure collapses the tumor blood vessels, limiting access by, and efficacy of, conventional forms of chemotherapy [60]. In genetically-engineered mouse models of pancreatic adenocarcinoma, which mimic the clinical syndrome, histopathology, and molecular progression of the human disease, combined enzymatic degradation of hyaluronan and cytotoxic therapy leads to a rapid reduction of the IFP, accompanied by remodeling of the standard cytotoxic delivery; this combined approach thus holds promise as a therapeutic strategy [61].

On the basis of these studies, and in consideration of the complex role of tumor vascularization in relations between chemotherapic drugs and tumor cells, treatment approaches involving targeting angiogenesis and vasculogenesis should be evaluated with extreme care.

## Conclusions

This study demonstrates the ability of pancreatic carcinoma cells to attract CD34^+^ cells to the tumor site, and to interfere with those cells' hematopoietic development, by diverting their differentiation toward endothelial cells; these cells can become a component of the tumor vasculature. A better understanding of cancer-associated molecular mechanisms involved in favoring the vasculogenesis process in pancreatic carcinoma could therefore provide important knowledge to design new and more effective ways of combining anti-angiogenic drugs with established chemotherapies, the goal being to prevent angiogenic escape or invasion.

## Abbreviations

VEGF: Vascular endothelial growth factor; EPC: Endothelial progenitor cells; BM: Bone marrow; Angio: Angiopoietin; FCS: Fetal calf serum; CM: Conditioned medium; HUVEC: Human umbilical vein endothelial cells; IL: Interleukin; SCF: Stem cell factor; GM-CSF: Granulocyte-macrophage colony stimulating factor; LSCM: Laser scanning confocal microscopy; Dil-AcLDL: 1,1-dioctadecyl-3,3,3,3-tetramethilindocarbocyanine-labeled acetylated low-density lipoprotein; FITC: Fluorescein isothiocyanate.

## Competing interests

The authors declare that they have no competing interests.

## Authors' contributions

BV carried out all flow cytometry, real time RT–PCR, and LSCM analyses, participated in data analysis, and helped to draft several chapters of the manuscript; FB provided expertise on LSCM study and was involved in the discussion of the results and manuscript preparation; TS performed immunomagnetic separation and cultures of CD34^+^ cells, AN carried out statistical analysis of the data; AP was involved in cytospin preparations and IHC analysis; LC, GM, GP participated in discussion of the data and preparation of the manuscript; GB conceived of and designed the study, coordinated the work, carried out experiments on Matrigel, and prepared the manuscript. All authors read and approved the final manuscript.
